# Supplementary material for: ProteinShader: illustrative rendering of macromolecules
Source: BMC Struct Biol. 2009 Mar 30;9:19. doi: 10.1186/1472-6807-9-19 (PMC2672931; doi:10.1186/1472-6807-9-19)
Supplement: Additional file 1 — ProteinShader program without source code. This compressed file contains the complete ProteinShader program including associated libraries, but no source code. A README.txt file gives an overview of the ProteinShader distribution, and the index.html file in the help subdirectory has directions on getting started with the program as well as a set of tutorials. [file 1472-6807-9-19-S1.zip › ProteinShader-beta-0_9_4-binary/help/api/org/proteinshader/structure/enums/package-summary.html]

org.proteinshader.structure.enums (ProteinShader API)


|  |  |  |
| --- | --- | --- |
| **Overview** \| **Package** \| Class \| **Use** \| **Tree** \| **Deprecated** \| **Index** \| **Help** | |  |
| **PREV PACKAGE**   **NEXT PACKAGE** | **FRAMES**    **NO FRAMES**     **All Classes** |  |


---

## Package org.proteinshader.structure.enums

Holds the enums needed by org.proteinshader.structure and its subpackages.

**See:**
  
          **Description**

| **Enum Summary** | |
| --- | --- |
| **AAColorEnum** | Provides an enumeration of an amino acid color scheme. |
| **AminoAcidEnum** | Provides an enumeration of the amino acids found in protein. |
| **AtomEnum** | Provides an enumeration of atom types: C, H, N, O, S, *etc.* |
| **BondEnum** | Provides an enumeration for molecular bonds. |
| **CPKColorEnum** | Provides an enumeration of the CPK color scheme that is needed by AtomEnum. |
| **DecorationEnum** | Provides an enumeration to specify if a Segment object should be plain, with text labels, or with halftoning when the Segment is rendered as a three-dimensional tube or ribbon. |
| **DrawableEnum** | Provides an enumeration to specify if a Drawable object is an Atom, Bond, or Segment. |
| **HelixEnum** | Provides an enumeration of the helix types specified in the  Protein Data Bank . |
| **HelixShapeEnum** | Lists the possible shapes that can be used to represent a Helix. |
| **RegionColorEnum** | PrProvides default colors for regions of protein secondary structure (alpha-helices, beta-strands and general loop regions). |
| **RegionEnum** | Provides a Region type enumeration (Loop, Helix, or BetaStrand) useful for marking AminoAcids. |
| **ResidueEnum** | Provides an enumeration that can be used to restrict a Visitor to traversing only a particular Residue type. |
| **SideChainEnum** | Provides an enumeration to specify how an amino acid side chain should be displayed is it is shown in combination with a tube or ribbon segment. |
| **VisibilityEnum** | Provides an enumeration to specify if a Drawable object is opaque, translucent, or invisible. |

## Package org.proteinshader.structure.enums Description

Holds the enums needed by org.proteinshader.structure and its subpackages.
  
  

## Related Documentation

For help and tutorial pages, please see:

- http://proteinshader.sourceforge.net

**Version:**
:   Beta 0.9.4 (December 30, 2008)

**Author:**
:   Joseph R. Weber

---


|  |  |  |
| --- | --- | --- |
| **Overview** \| **Package** \| Class \| **Use** \| **Tree** \| **Deprecated** \| **Index** \| **Help** | |  |
| **PREV PACKAGE**   **NEXT PACKAGE** | **FRAMES**    **NO FRAMES**     **All Classes** |  |


---

# *Copyright © 2007-2008*
